# Assessment of the Impact of the COVID-19 Pandemic on the Pro-Health Behavior of Poles

**DOI:** 10.3390/ijerph19031299

**Published:** 2022-01-24

**Authors:** Karolina Hoffmann, Anna Paczkowska, Aleksandra Bońka, Michał Michalak, Wiesław Bryl, Dorota Kopciuch, Tomasz Zaprutko, Piotr Ratajczak, Elżbieta Nowakowska, Krzysztof Kus

**Affiliations:** 1 Department of Internal Diseases, Metabolic Disorders and Arterial Hypertension, Poznan University of Medical Sciences, Szamarzewskiego 82/84 Street, 60-101 Poznań, Poland; karhof@o2.pl (K.H.); wieslawbryl@wp.pl (W.B.); 2 Department of Pharmacoeconomics and Social Pharmacy, Poznan University of Medical Sciences, Rokietnicka 7 Street, 60-806 Poznań, Poland; ola.bonka@wp.pl (A.B.); dorota.koligat@gmail.com (D.K.); tomekzaprutko@ump.edu.pl (T.Z.); p_ratajczak@ump.edu.pl (P.R.); kkus@ump.edu.pl (K.K.); 3 Department of Computer Science and Statistics, Poznan University of Medical Sciences, Rokietnicka 7 Street, 60-806 Poznań, Poland; michal@ump.edu.pl; 4 Department of Pharmacology and Toxicology, Institute of Health Sciences, Collegium Medicum, University of Zielona Góra, Licealna 9 Street, 65-417 Zielona Góra, Poland; elapharm@ump.edu.pl

**Keywords:** COVID-19, SARS-CoV-2 pandemic, pro-health behavior

## Abstract

The SARS-CoV-2 virus pandemic has exerted enormous impacts on individuals’ lifestyle, economics and social life. The aim of the study was to assess the impact of the COVID-19 pandemic on health-promoting behaviors of a sample of the Polish population, including dietary supplementation, physical activity, eating habits, and the implementation of preventive vaccinations. Within the scope of a survey, data was collected from 1101 adult respondents residing in Poland (862 women and 239 men). An anonymous questionnaire prepared based on the scientific literature was used as a research tool. The survey was conducted during the second wave of COVID-19 using the CAWI (Computer—Assisted Web Interview) method by disseminating the questionnaire using selected social media. We found that, during the COVID-19 pandemic, the respondents were more likely to maintain healthy eating habits and use dietary supplementation; however, the majority engaged in physical activity less frequently and had a sedentary lifestyle. Most felt no need to undergo preventive influenza vaccinations. The COVID-19 pandemic significantly increased the health awareness of the analyzed group from the Polish population. However, the obtained results are not fully satisfactory; therefore, there is a need to promote a healthy lifestyle and pro-health behaviors as part of social campaigns.

## 1. Introduction

COVID-19 is an infectious disease caused by a new coronavirus discovered in 2019, initially referred to as 2019-nCoV [1]. On 11 February 2020, the International Committee on Taxonomy of Viruses (ICTV) gave the virus its official name: SARS-CoV-2 [2]. The first COVID-19 patient was confirmed by the World Health Organization (WHO) on 8 December 2019, in Wuhan city, the capital of Hubei province in central China. As of 20 January 2020, 278 confirmed cases of 2019-nCoV had been reported from China [3]. On 13 January 2020, the first patient outside of China—in Thailand—was diagnosed. At the end of that same month, the new coronavirus reached Europe with two patients diagnosed in France [4]. 

On 30 January 2020, WHO declared the COVID-19 outbreak a ‘Public Health Emergency of International Concern (PHEIC), and then on 11 March 2020, a pandemic was announced, when four countries (Japan, South Korea, Iran, and Italy) reported a rapid increase of cases [5,6]. During the second wave of the COVID-19 pandemic in Poland, more than 1.4 million diagnosed cases were reported; of these, 31,089 people had died [7]. 

Restrictions imposed by the Polish government changed in relation to the numbers of daily new infections and related deaths. These included travel restrictions and the mandatory closure of schools, nonessential commercial activities, and industries in most cases. People were asked to stay at home and socially isolate themselves to prevent contracting an infection [8]. The pandemic has had far-reaching impacts, affecting global politics, economy, culture, public health, and many other areas, including social life and health-promoting behaviors [9]. 

According to a study performed by Makowska et al., adherence to the restrictions imposed by the government was declared by 60.6% of Poles during the second waves of the COVID-19 pandemic. A recommended behavior to which most respondents (80.3%) adhered was wearing a mask in every situation where the government recommended it. More than half of Poles (66.3%) were trying to better manage their immunity by engaging in appropriate healthy behavior. The rarest recommended behavior declared by Poles was the declaration to vaccinate against influenza (30.1%) [10].

A health-promoting behavior is any activity that leads to one maintaining or improving health [11]. As such, they are an essential part of preventing many diseases. According to the definition formulated by WHO, health is a state of complete physical, mental and social well-being and not merely the absence of disease or infirmity [12]. 

Due to this, health-promoting behavior should be understood as, among other things, healthy eating habits, physical activity, and giving up stimulants [11,13,14]. Research conducted thus far in Poland and in the world has shown the positive effect of the COVID-19 pandemic on health-promoting behavior in humans. The above behaviors have been manifested by an increase in the use of appropriate dietary supplements, such as vitamin C or D [15], and OTC drugs with anti-viral and immunostimulating properties [16], the use of a balanced diet [17], and adherence to the restrictions imposed by the government [10].

The primary objective of this research paper was to assess the impact of the COVID-19 pandemic on the health-promoting behavior of Poles, i.e., supplementation, physical activity, eating habits, and awareness of the need for vaccinations during the so-called second wave of the disease in Poland (1 November 2020–31 January 2021). The study also identified selected sociodemographic information (gender, age, education, place of residence, work activity status, and location of work during the pandemic), clinical data (health status, presence of comorbidities, and COVID-19 incidence), and environmental factors (risk of SARS-CoV-2 infection, direct contact with a person with a known coronavirus infection, and contact with a person with a suspected coronavirus infection or infectious materials) that statistically influence health-promoting behavior [18,19].

## 2. Materials and Methods

### 2.1. Sample Composition

The sample size was estimated based on the formula for a finite population [20]. Assuming that 20 million adult Poles have access to the Internet [21], the estimated fraction size 50% (standard if the fraction is unknown), the significance level of α = 0.05 and permissible error (e) = 3%, the necessary sample size is 1068. Ultimately, 1101 respondents (862 women and 239 men) took part in the study.

All respondents were of legal age under Polish law and resided in Poland. Survey participants satisfied the predefined inclusion criteria, i.e., at least 18 years of age, currently living in Poland, and willing to provide informed consent to participate in the study. 

Individuals who did not meet the inclusion criteria were excluded from the survey. The survey process began following an opinion issued on 4 November 2020 by the Bioethics Committee at the Poznan University of Medical Sciences confirming that the study did not have the features of a medical experiment. Before taking the survey, each participant was made aware of the purpose of the research and was informed that the study was safe, accessible, and anonymous. The consent to participate in the study could be withdrawn at any time without giving any reason.

### 2.2. Research Methods and Time Horizon

A bespoke anonymous questionnaire on Google Forms based on the scientific literature was used as a research tool. The survey was conducted between 1 November 2020 and 31 January 2021. Due to the pandemic and the following restrictions, to avoid any potential risk to the survey subjects, the survey was implemented using the CAWI (Computer—Assisted Web Interview) method. Social media was used to distribute questionnaires. Questionnaires were distributed electronically throughout the country. 

The questionnaire comprised 44 questions divided into six sections, which included: respondent particulars, respondents health condition evaluations during the pandemic, analysis of dietary supplementation used by respondents, assessment of the physical activity of respondents during the pandemic, evaluation of respondent lifestyles during the pandemic, analysis of prevalence, and course of COVID-19 among Polish respondents (the questionnaire is reprinted in Supplementary Material File S1).

### 2.3. Statistical Analysis

Statistical analysis was applied to the obtained results. The analyzed measurable parameters were represented using the mean, median, and standard deviation values, while the non-measurable parameters used counts and percentages. Results between the studied groups were compared using the Student’s *t*-test. A comparison was made using the Mann–Whitney test where data did not follow a normal distribution (Shapiro–Wilks test). More than two groups were compared using the Kruskal–Wallis test with Dunn’s post hoc test. 

For unrelated qualitative characteristics, the chi-homogeneity test was used to detect the existence of differences between compared groups. The relationship between the selected health-promoting behaviors and the analyzed parameters was carried out using multiple and logistic regression analysis. The analysis was performed using the TIBCO Software Inc. (2017) statistical package. Statistica (data analysis software, TIBCO Software Inc., Palo Alto, CA, USA, ver. 13.3 http://statistica.io (accessed on 30 March 2021; the date of the next licence for Poznan University of Medical Sciences from 31 October 2021). A test probability of *p* < 0.05 was assumed to be significant.

## 3. Results

### 3.1. Sample Characteristics

The sample consisted of 1101 adult respondents, including 862 women and 239 men. 45.59% of respondents were in the 18–24 age group, 33.06% of respondents were aged 25–40, 16.08% of respondents were between 41 and 60 years old, and 5.27% of respondents were over 60. Each participant satisfied the predefined inclusion and exclusion criteria. The mean body weight of the respondents was 68.22 ± 14.91 kg (median—65 kg). The mean height of the respondents was 169.52 ± 8.32 cm (median—169 cm). The mean Body Mass Index (BMI) was 23.75 ± 6.40 kg/m^2^ (median—22.77 kg/m^2^). The vast majority of respondents declared that their health status was good (43.14%) or very good (41.51%). It was described as excellent by 7.90% of respondents, as not very good by 6.36% and as poor by 1.09% of respondents. See Table 1 for a detailed specification of the sample.

### 3.2. Analysis of Health-Promoting Behaviors of Poles during the Second Wave of the COVID-19 Pandemic

#### 3.2.1. Dietary Supplementation

According to the research, 55.86% of the study population used dietary supplements during the second wave of the COVID-19 pandemic. The most common were vitamin D3 (45.96%), vitamin C (31.43%), magnesium (25.52%), and B vitamins (18.44%) (Table 2).

Most respondents spent similar amounts of money per month on dietary supplements during the COVID-19 pandemic as they did before the pandemic (74.47%). Spending on supplementation during the pandemic increased for 22.93% of respondents. A total of 2.60% of respondents reported spending less on dietary supplements during the pandemic.

#### 3.2.2. Eating Habits

The majority of respondents attempted to eat healthily during the COVID-19 pandemic. More than half of the respondents avoided fizzy drinks (57.31%), attempted to limit their consumption of sweets (54.41%), and drank at least 1.5 L of water a day (53.41%). A sizeable proportion of the sample population consumed plenty of fruit and vegetables (50.05%), regularly ate (45.14%), and ensured that their diet was balanced (41.60%). 

The vast majority of the respondents did not change their diet under the impact of the COVID-19 pandemic (67.67%), while 19.62% of the respondents attempted to eat more healthily. We found that 2.91% of the respondents gave up stimulants during the pandemic, and 11.35% of respondents changed their diet to a less healthy one due to the pandemic. An overall assessment of dietary habits in the sample showed that most respondents had three healthy nutritional habits (21.98%) (Table 3).

#### 3.2.3. Physical Activity

The most significant group of respondents (38.06%) did not engage in any physical activity during the COVID-19 pandemic. The exact number of respondents involved in physical activity once a week and two to three times a week (26.43% each). Only 9.08% of the respondents were physically active more than three times a week. The vast majority of Poles subject to the survey (54.32%) declared that the COVID-19 pandemic had not affected their physical activity in any way. In comparison, 30.97% of the respondents admitted that the current epidemic situation had reduced their level of physical activity. Only 14.71% of the respondents were physically active more than before.

#### 3.2.4. Vaccinations

The survey results showed that, during the pandemic (2020), the vast majority of the respondents did not vaccinate against influenza because they did not feel the need to do so (66.94%). A sizeable proportion of the respondents did not receive influenza vaccination due to a lack of vaccine (19.62%). Individuals who received a flu vaccination in 2020 but did not do so in 2019 accounted for 5.63% of the respondents. Only 5.27% of the respondents had the above vaccination administered in 2019 and 2020. In contrast, 2.54% of those surveyed had a flu vaccination only in 2019.

### 3.3. Logistic Regression Model of the Influence of Selected Clinical, Sociodemographic and Environmental Factors on the Adherence to Health-Promoting Behaviors of Poles during the Second Wave of the COVID-19 Pandemic

#### Dietary Supplementation

Logistic regression analysis showed that factors, such as age (*p* = 0.011), risk of coronavirus infection at work (*p* < 0.001), health status (*p* = 0.018), back pain (*p* = 0.010), depression (*p* = 0.036), and COVID-19 incidence (*p* = 0.009) significantly influenced the frequency of dietary supplementation use among the respondents. A linear regression model showed that the oldest age group of respondents (over 60) was statistically significantly more likely to use dietary supplements. A similar trend was observed for professionally active people. 

The greater the risk of a SARS-CoV-2 infection in the workplace, the more significant the role of supplementation in prevention among the respondents. Survey participants in poorer health were more likely to use dietary supplements. Respondents who suffered from back pain and depression were significantly more likely to take dietary supplements. A statistically significant increase in the levels of supplementation was also found for those with a past SARS-CoV-2 infection (Table 4).

### 3.4. Eating Habits

A logistic regression model showed that age had a statistically significant effect on having healthy eating habits (*p* = 0.037). As their age increased, the respondents ate more nutritious diets. The presence of a statistically significant relationship between health status and the number of healthy eating habits was demonstrated (*p* < 0.001). Respondents who did not follow a healthy diet exhibited poorer health. Allergy sufferers were statistically significantly more likely to have more healthy eating habits than the other patient groups subject to the study (*p* = 0.017).

### 3.5. Physical Activity

According to a logistic regression model, the level of physical activity among respondents decreased significantly with age (*p* = 0.005). The pupil/student population exhibited the highest statistically significantly level of physical activity (*p* = 0.005) out of the entire sample. It was demonstrated that the worse the health status of the respondents, the less physical activity the respondents engaged in (*p* < 0.001). Similarly, the occurrence of diseases, such as high blood pressure (*p* = 0.001), degenerative spine disease (*p* = 0.003), and respiratory diseases (*p* = 0.045), had a statistically significant impact on the reduction of physical activity levels among respondents (Table 4).

### 3.6. Vaccinations

Logistic regression analysis demonstrated that having an influenza vaccination was associated with age (*p* < 0.001), place of residence (*p* < 0.001), and work activity status (*p* < 0.001) of the respondents. Respondents over 60 years of age, living in cities of more than 250,000 residents, and receiving pension benefits were much more likely to be vaccinated against influenza compared to other survey participants. 

A linear regression model also confirmed the impact of the risk of a COVID-19 infection in the workplace (*p* = 0.044) and the presence of some chronic diseases, such as coronary artery disease/prior heart attack (*p* = 0.023), high blood pressure (*p* = 0.003), osteoarthritis (*p* < 0.001), back pain (*p* < 0.001), allergies (*p* = 0.006), and depression (*p* = 0.017) on influenza vaccinations. Respondents who worked in areas with a substantial risk of coronavirus infection or had the aforementioned medical conditions were significantly more likely to opt for an influenza vaccination (Table 4).

## 4. Discussion

At the beginning of discussing the obtained results, it is worth emphasizing that the presented work is innovative and constitutes a valuable compendium of scientific knowledge on the impact of the COVID-19 pandemic on changes to the health-promoting behavior of Poles. The detailed statistical data obtained relates to dietary supplementation, physical activity, eating habits, and immunization.

Research shows that, during the COVID-19 pandemic, the role of health prevention and healthy lifestyles in the Polish society increased significantly. The vast majority of respondents subject to the survey used dietary supplementation (55.86%). The age of the respondents significantly influenced the increased use of dietary supplements, risk of coronavirus infection in the workplace, health status, certain chronic diseases (back pain and depression), and COVID-19 incidence. It is worth noting that, for as many as 22.93% of the survey respondents, the monthly expenditure on dietary supplements increased with the amount spent on this purpose before the pandemic. This represents almost a quarter of the surveyed population sample. We found that 74.47% of respondents declared that their spending on dietary supplements did not change due to the coronavirus pandemic, while only 2.60% of respondents estimated that the monthly amount spent on supplementation now was less than before the pandemic. 

A Polish study by Gardocka-Jalowiec et al. assessed the impact of the SARS-CoV-2 virus pandemic on the consumption of OTC (over the counter) drugs in Poland [16]. Based on monthly January–June data (volume and value) between 2017 and 2020 for OTC drug sales, it was estimated that 2020 was a record year for OTC drug sales. In March 2020, when the first case of COVID-19 was diagnosed in Poland, more than 126 million OTC drugs were sold. This compares to just under 85 million OTC drugs sold in March 2019.

An analysis of the use of dietary supplements during the coronavirus pandemic using the Google Trends tool and the PLifeCOVID-19 online survey was also carried out by Hamulka et al. [22]. An investigation of search phrases on the Google platform positively correlated with COVID-19 showed an increased interest in immune-enhancing products in Poland and worldwide. Poland’s most frequently searched dietary supplements were vitamin D3, vitamin C, honey, iron, and magnesium; globally, the most popular searches were for magnesium, vitamin D3, iron, honey, and vitamin B12. 

These conclusions were confirmed by a survey conducted during the first and second waves of the coronavirus pandemic. Hamulka et al. [22] estimated that 48% of the respondents who took part in the survey in April/May 2020 took dietary supplements. In November of the same year, 79% of surveyed respondents took dietary supplements. The cited data are consistent with the results obtained in this study. According to the PLife COVID-19 questionnaire responses, vitamin D3 (38% of respondents during the first and 67% during the second wave) and vitamin C (17% of respondents during the first and 37% during the second wave) were supplemented most frequently. 

This paper shows that Polish respondents were also most likely to indicate vitamin D3 and vitamin C as dietary supplements (45.96% and 31.43% of respondents, respectively). The obtained results are consistent with the study performed by Puścion-Jakubik et al. [15]. Researchers from the Medical University of Bialystok explored the problem of food supplement intake, especially zinc and vitamin D, during the COVID-19 pandemic in Poland. Both ingredients were used significantly more often by participants with higher education (59.0%), with a medical background or with related work in the medical field (54.5%), and/or exercising at home (60.1%). Vitamin D was taken by 22.8% of the respondents in the first wave, 37.6% in the second wave, and 32.9% in the third wave [15].

The COVID-19 pandemic also undoubtedly affected the eating habits of respondents of different nationalities. A study by Ghosh et al. found increased carbohydrate consumption and increased snacking between meals among type 2 diabetes patients in northern India (21% and 23% of respondents, respectively) [23]. However, as many as 27% of those surveyed said they had also consumed more fruit during the coronavirus pandemic. In the present study, 50.05% of the respondents stated that they consumed many fruits and vegetables. 

A survey carried out by Di Renzo et al. on the Italian population assessed the direct impact of the SARS-CoV-2 virus pandemic on lifestyle change [24]. A considerable proportion of respondents reported following a healthy Mediterranean diet and buying fresh fruit and vegetables directly from organic farms under the influence of the pandemic. On top of that, 3.3% of those surveyed had decided to give up smoking. In the present paper, more than half of the respondents avoided fizzy drinks (57.31%), attempted to limit their consumption of sweets (54.41%), and drank at least 1.5 L of water a day (53.41%). Only 2.91% of the respondents gave up stimulants due to the coronavirus pandemic.

A study by Górnicka et al. demonstrated that the COVID-19 pandemic had an ambiguous effect on the dietary habits of Poles [17]. An analysis of the data revealed that 19% of those surveyed consumed more unhealthy foods during the pandemic. At the same time, a considerable proportion of respondents declared that they ate healthy home-cooked meals more often and attempted to choose high-quality foods. A total of 53% of the respondents did not change their diets. 

The present study shows that 19.62% of the surveyed Poles attempted to eat healthier diets, 11.35% of the respondents changed their diet to a less favorable one, and, for 67.67% of the survey participants, the pandemic did not bring about a change in diet. The results of both studies confirmed that older respondents were statistically significantly more likely to eat healthier diets than the younger part of the population sample.

The obtained data lead to a conclusion that the COVID-19 pandemic has made many Poles aware of the health importance of a properly balanced diet and consumption of high-quality food products as well as carefully selected dietary supplementation. The obtained results are confirmed in the scientific literature. According to a study performed by Makowska et al., more than half of Poles (66.3%) were attempting to better manage their immunity by engaging in appropriate healthy behavior [10]. It was demonstrated that nutritional status directly affects the functioning of the immune system and susceptibility to disease [25]. The present study showed a relationship between health status and diet amongst the surveyed Poles. Respondents with unhealthy eating habits had a significantly poorer health status. This was also confirmed in a paper by Naja et al. [25]. 

In their article, Yu X. et al. highlighted that a change in health-promoting behavior was reflected in a reduced hospitalization rate for certain diseases. During the COVID-19 pandemic, there was a reduced risk of metabolic diseases, such as acute pancreatitis, hyperlipidemia, and acute cholecystitis [26]. However, one should remember that diet deteriorated significantly for a significant percentage of the Poles surveyed. These findings illustrate the need for the government to develop public campaigns to promote healthy eating and programs to increase access to nutrition education, such as open helplines or newsletters.

In the present survey, 38.06% of the respondents did not engage in any physical activity during the COVID-19 pandemic. The same proportion of respondents involved in physical activity once a week and two to three times a week (26.43% each). Only 9.08% of the respondents engaged in physical activity more than three times a week. Such results are far from satisfactory. According to current WHO recommendations, adults should be active on most days of the week [27]. 

The recommended moderate-intensity aerobic physical activity duration is at least 150–300 min or 75–150 min for high-intensity exercises. It is also advisable to do muscle-strengthening exercises at least two days a week [27,28]. It is emphasized that regular physical activity significantly reduces the risk of many chronic diseases (including hypertension and type 2 diabetes), improves sleep quality, and positively impacts mental health [29]. 

Such findings are confirmed by the logistic regression analysis conducted in this study, which shows that respondents who exercise less frequently are characterized by statistically significantly worse health status. Statistical analysis also proved a relationship between decreased physical activity and chronic diseases, such as hypertension, degenerative spine disease, and respiratory diseases. Furthermore, it was found that younger respondents and pupils/students were statistically significantly more likely to engage in physical activity compared to other age and occupational groups.

In addition, data collected during this survey showed that as many as 30.97% of surveyed Poles were less likely to engage in physical activity due to the COVID-19 pandemic. A small proportion of respondents reported that their level of physical activity had increased (14.71%), while for 54.32% of respondents, the coronavirus pandemic did not affect the frequency of physical activity. Szczygielska et al. compared the frequency of different forms of physical activity currently and before the pandemic by people working remotely during the pandemic [30]. A two percent increase was observed in the proportion of respondents who exercised daily. With 42% of respondents exercising several times a week before the pandemic, only 30% of respondents remained physically active at the frequency mentioned above. Similarly, the proportion of those exercising once a week decreased from 15% to 9% of respondents. During the SARS-CoV-2 pandemic, a higher proportion of respondents engaged in physical activity sporadically or not at all compared to before the pandemic (9% and 8% more, respectively). 

The quoted results are consistent with the data collected in this paper. A study by Flanagan et al. estimated that respondents spent less (statistically significantly) time on physical activity during the COVID-19 pandemic [31]. During a national quarantine, respondents were significantly more likely to suffer from anxiety disorders and gain weight faster. Reduced physical activity among the sample population may be related to restrictions in the operation of sports facilities and the closure of gyms for nearly a year.

During the second wave of the COVID-19 pandemic in Poland, one of the recommendations of the medical board of the Ministry of Health was to vaccinate against influenza, which was to alleviate the course of coronavirus infection [32]. According to the 2020 CBOS (Centre for Public Opinion Research) survey on Poles’ attitudes towards COVID-19 and influenza vaccination, the percentage of people vaccinated against influenza in 2020/2021 did not change compared to 2016/2017 and amounted to 6% of the surveyed population [33]. On the other hand, the proportion of respondents saying they would like to be vaccinated against influenza increased from 7% in 2016 to 14% in 2020. We found that 81% of the surveyed Poles have not been vaccinated and do not intend to be vaccinated. According to the CBOS report, respondents who have been vaccinated against influenza during the current flu season are most likely to be over 65 years of age (10%) and with higher education (8%). In the sample population, willingness to be vaccinated against influenza was declared by the oldest respondents (19%), as well as residents of the largest cities (21%), and those with university education (17%) and primary/secondary education (16%) also had the highest proportion [33]. 

In this survey, 66.94% of respondents declared that they did not intend to be vaccinated against influenza. A total of 19.62% of the respondents expressed a willingness to have an influenza vaccine. Respondents who had been vaccinated against influenza accounted for 10.90%, out of which 5.27% also had an influenza vaccine last season. We found that 2.54% of the respondents chose not to get an influenza vaccination this season, despite having been vaccinated the year before. However, this means that more than twice as many people in the 2020/2021 flu season saw the need for immunization (5.63%) compared to respondents who opted out of these vaccinations despite having previously received an influenza vaccine (2.54%). 

Nevertheless, this result is not satisfactory. Still too few Poles recognize the need for immunization. The sample population that was significantly more likely to be vaccinated against influenza were patients over 60 years of age, residents of cities with more than 250,000 inhabitants, pensioners, people working in a place with a high risk of a COVID-19 infection, and those suffering from certain chronic diseases (coronary artery disease/prior heart attack, high blood pressure, osteoarthritis, back pain, allergies, and depression). This may stem from the fact that the elderly, people suffering from more illnesses, and those living in large cities are at a greater risk of an influenza virus infection, as well as a more severe course of the disease when co-morbidities are present. 

The obtained results are confirmed in the scientific literature. According to a study performed by Makowska et al., the rarest recommended behavior declared by Poles was the declaration to vaccinate against influenza (30.1%) [10]. The authors of that study also aimed to assessed the impact of trust in medicine on Polish citizens’ adherence to recommended behaviors during the second wave of COVID-19. They proved that 27.1% of respondents expressed low trust, 36.7% expressed moderate trust, and 36.3% expressed high trust. It was also showed that 15.8% of respondents had low adherence, 38.2% had moderate adherence, and 46.0% had high adherence to the healthcare experts’ recommendations. It can be expected that patients convinced of the validity of influenza vaccination will be more likely to get vaccinated against a new viral disease—COVID-19. 

Wawrzuta D. et al. aimed to characterize the arguments against COVID-19 vaccines seen on Facebook in Poland [34]. They indicated that the main concern was the classic argument about the negative attitude of citizens to government vaccination campaigns. These studies also found that comments about vaccinations had overwhelmingly negative sentiment, but the responses were positive, suggesting that pro and anti-vaccine groups had different coping patterns with social media content [34].

Sakamoto et al. estimated that influenza activity during the COVID-19 pandemic (2020) in Japan was significantly lower compared to previous years [35]. The authors hypothesized that this may be a result of the daily use of personal protective equipment to reduce SARS-CoV-2 virus transmission and increased public awareness of infectious diseases. A Polish CBOS survey showed that currently 65% of Poles have a positive attitude towards COVID-19 vaccination (data as of April 2021), out of which 21% had already taken at least one dose of a COVID-19 vaccine, and the rest expressed willingness to be vaccinated [36]. Based on post-November 2020 observations, this trend is steadily increasing.

### Study Limitations and Strengths

The most important limitation is the fact that the sample structure does not reflect the gender structure in the general population of Polish nationality. More women (78%) took part in the survey, which results from the more significant activity of women in social media. Another limitation is the online research, which results from the current epidemiological situation and restrictions imposed by the government during the second wave of the pandemic. Moreover, online research does not provide a random nature of recruitment limited by potential respondents’ access to the internet or the respondents’ interest in the topic under study. 

On the other hand, the structure of the online questionnaires (most of the mandatory fields) did not allow for omitting the answers, which is more often observed when filling out paper questionnaires. However, this paper is a reliable and comprehensive source of knowledge in terms COVID-19 pandemic data and can therefore meet the extensive information needs of health care decision-makers, epidemiologists, and the wider medical community (including doctors, pharmacists, nutritionists, physiotherapists, and psychologists). The paper also contains a call for better health awareness addressed to representatives of the media at large as well as suggestions for themes of social programs and campaigns.

## 5. Conclusions

The COVID-19 pandemic significantly increased the health awareness of the analyzed group of the Polish population. The surveyed Poles were more likely to maintain healthy eating habits and use dietary supplements. The obtained results are still not satisfactory, and therefore educational activities in the field of lifestyle medicine and the dissemination of scientific evidence demonstrating the link between an appropriate lifestyle and health are needed.

## Figures and Tables

**Table 1:** Sociodemographic and clinical characteristics of the surveyed Poles (*n* = 1101).

Variables	Answers	Number of Respondents [*n*]	Percentage of Respondents [%]
Sex	female	862	78.29
	male	239	21.71
Age	18–24	502	45.59
	25–40	364	33.06
	41–60	177	16.08
	more than 60	58	5.27
Education	primary	6	0.54
	middle school	11	1.00
	basic vocational	30	2.72
	high school	428	38.87
	university	626	56.86
Place of residence	village	290	26.34
	town, up to 50 thousand residents	179	16.26
	town, up to 100 thousand residents	147	13.35
	city, up to 250 thousand residents	64	5.81
	city, more than 250 thousand residents	421	38.24
Employment status	pupil/student	430	39.06
	professionally active	574	52.13
	unemployed	48	4.36
	pensioner	47	4.27
	on benefits	2	0.18
Type of professional work	blue-collar	132	23.00
	white-collar	442	77.00
Place of work during the pandemic	at a workplace	405	36.78
	remote work from home	152	13.80
	unemployed	18	1.63
Chronic diseases	obesity	55	5.00
	coronary artery disease/prior myocardial infarction	8	0.73
	arterial hypertension	82	7.45
	atherosclerosis	4	0.36
	stroke	3	0.27
	diabetes	20	1.82
	osteoarthritis	28	2.54
	spondylarthritis	56	5.09
	back pains	126	11.44
	rheumatic disorder	15	1.36
	allergy	178	16.17
	cancer	10	0.91
	respiratory system diseases	37	3.36
	depression	56	5.09
	no diagnosed diseases	590	53.59
	other	197	17.89

**Table 2:** Dietary supplements taken by the respondents during the second wave of the COVID-19 pandemic in Poland.

Dietary Supplement	Number of Respondents [*n*]	Percentage of Respondents [%]
vitamin D_3_	506	45.96
vitamin C	346	31.43
group B vitamins	203	18.44
omega-3 acids	147	13.35
zinc	138	12.53
magnesium	281	25.52
iron	103	9.36
potassium	9	0.82
selenium	11	1.00
calcium	5	0.45
vitamin A	5	0.45
vitamin E	5	0.45
vitamin K	8	0.73
vitamin complex	25	2.27
mineral complex	6	0.54
Ashwagandha	6	0.54
other	54	4.90

**Table 3:** Analysis of dietary habits for Poles during the COVID-19 pandemic.

Eating Habits	Number of Respondents [*n*]	Percentage of the Respondents [%]
I eat a lot of fruits and vegetables	551	50.05
I eat 5 meals every day	315	28.61
I eat regularly	497	45.14
I drink at least 1.5 L of water a day	588	53.41
I am trying to cut down on sweets	599	54.41
I avoid sparkling soft drinks	631	57.31
I do my best to eat a balanced diet	458	41.60
I eat a lot of fast foods	94	8.54
none of the above	52	4.72
Number of healthy eating habits *		
−1	16	1.45
0	70	6.36
1	110	9.99
2	188	17.08
3	242	21.98
4	198	17.98
5	147	13.35
6	88	7.99
7	42	3.81

* on the basis of the question about the respondents’ eating habits, the number of eating habits for each individual was calculated, assuming the following relationships: for the answer “I eat a lot of fast-food” a point was subtracted, for the answer “none of the above” no points were given, and for the remaining answers, one point was awarded, thus, obtaining a real view of how the respondents ate.

**Table 4:** Logistic regression analysis of selected factors associated with dietary supplementation, adherence to healthy eating habits, physical activity and influenza vaccination among Poles during the “second wave” of the COVID-19 pandemic in Poland.

Variable	Dietary Supplementation			Number of Healthy Eating Habits			Physical Activity			Influenza Vaccination		
	OR	[95% CI]	*p*	OR	[95% CI]	*p*	OR	[95% CI]	*p*	OR	[95% CI]	*p*
**Sex**												
female	1.0 → (ref)			1.0 → (ref)			1.0 → (ref)			1.0 → (ref)		
male	0.76	[0.57, 1.02]	0.066	0.18	[−0.60, 0.95]	0.653	1.29	[0.95, 1.74]	0.1	0.68	[0.41, 1.14]	0.144
**Age**												
18–24	1.0 → (ref)			1.0 → (ref)			1.0 → (ref)			1.0 → (ref)		
25–40	1.42	[1.08, 1.87]	**0.011**	0.65	[−0.14, 1.44]	0.106	0.67	[0.51, 0.89]	**0.005**	1.3	[0.83, 2.04]	0.257
41–60	1.3	[0.92, 1.83]	0.141	1	[0.06, 1.93]	**0.037**	0.81	[0.57, 1.15]	0.235	1.04	[0.57, 1.88]	0.899
more than 60	1.81	[1.03, 3.20]	**0.041**	1.71	[−0.15, 3.56]	0.071	0.58	[0.33, 1.00]	0.05	3.94	[2.04, 7.64]	**<0.001**
**Education**												
middle school	1.0 → (ref)			1.0 → (ref)			1.0 → (ref)			3.88	[0.69, 21.91]	0.125
primary	6.00 am	[0.52, 69.75]	0.152	−0.2	[−2.19, 1.79]	0.845	0.19	[0.02, 1.62]	0.128	2.22	[0.84, 5.84]	0.107
basic vocational	1.2	[0.30, 4.80]	0.797	0.17	[−0.55, 0.89]	0.641	0.38	[0.08, 1.69]	0.202	1.32	[0.87, 2.00]	0.196
high school	1.16	[0.35, 3.85]	0.813				0.59	[0.15, 2.26]	0.443			
university	1.85	[0.56, 6.14]	0.312				0.64	[0.17, 2.44]	0.515			
**Place of residence**												
village	1.0 → (ref)			1.0 → (ref)			1.0 → (ref)			1.0 → (ref)		
town, up to 50 thousand residents	0.78	[0.53, 1.13]	0.186	0.74	[−0.17, 1.66]	0.11	0.78	[0.53, 1.13]	0.185	1.12	[0.56, 2.23]	0.755
town, up to 100 thousand residents	0.68	[0.46, 1.01]	0.058	0.36	[−0.63, 1.34]	0.478	1.05	[0.70, 1.58]	0.804	1.2	[0.58, 2.47]	0.624
city, up to 250 thousand residents	1.57	[0.89, 2.78]	0.12	0.24	[−1.04, 1.52]	0.707	1.75	[0.97, 3.17]	0.064	1.2	[0.43, 3.33]	0.732
city, more than 250 thousand residents	1.11	[0.82, 1.51]	0.491	0.32	[−0.48, 1.11]	0.433	1.36	[1.00, 1.86]	0.05	2.53	[1.51, 4.24]	**<0.001**
**Employment status**												
pensioner	1.0 → (ref)			1.0 → (ref)			1.0 → (ref)			1.0 → (ref)		
professionally active	0.99	[0.55, 1.82]	0.986	0.95	[0.58, 1.77]	0.955	1.52	[0.84, 2.75]	0.171	0.22	[0.11, 0.43]	**<0.001**
unemployed	1.35	[0.59, 3.09]	0.476	1.19	[0.66, 2.88]	0.589	0.63	[0.28, 1.42]	0.262	0.08	[0.02, 0.36]	**0.001**
pupil/student	0.83	[0.45, 1.52]	0.544	0.77	[0.44, 1.32]	0.633	2.38	[1.30, 4.37]	**0.005**	0.27	[0.13, 0.53]	**<0.001**
on benefits	0.74	[0.04, 12.57]	0.835	0.76	[0.03, 11.44]	0.935						
**Place of work during the pandemic**												
Unemployed	1.0 → (ref)	[0.02, 2.60]	0.223	0.39	[−1.10, 1.89]	0.606	1.0 → (ref)	[0.52, 4.78]	0.421	1.24	[0.64, 2.40]	0.52
at a workplace	0.84	[0.13, 2.09]	0.364	0.93	[−1.05, 2.91]	0.356	1.63	[0.42, 6.36]	0.479			
remote work from home	0.93	[0.29, 2.98]	0.906	1.44	[−0.15, 3.03]	0.076	2.06	[0.66, 6.43]	0.215			
**COVID−19 infection risk at the workplace**												
yes, low	2.02	[1.04, 3.93]	**0.037**	0.34	[−1.05, 1.73]	0.631	1.37	[0.68, 2.73]	0.379	0.22	[0.02, 2.04]	0.184
yes, moderate	2.33	[1.27, 4.25]	**0.006**	0.89	[−0.44, 2.23]	0.189	0.92	[0.50, 1.68]	0.78	0.91	[0.24, 3.36]	0.883
yes, high	2.62	[1.54, 4.45]	**<0.001**	0.84	[−0.46, 2.14]	0.203	0.7	[0.41, 1.19]	0.192	2.99	[1.03, 8.67]	**0.044**
**Health status**												
excellent	1.0 → (ref)	[0.78, 1.95]	0.377	1.0 → (ref)	−2.45 [−0.21, 0.07]	**0.02**	1.0 → (ref)	[0.51, 1.47]	0.603	1.0 → (ref)	[0.94, 6.28]	0.068
very good	1.23	[1.00, 2.51]	0.05	−1.33	−3.55 [−1.28, 0.07]	**<0.001**	0.87	[0.24, 0.67]	**0.001**	2.43	[0.80, 5.44]	0.13
good	1.58	[1.12, 4.11]	**0.021**	−2.42	−3.77 [−0.53, 0.07]	**0.01**	0.4	[0.11, 0.45]	**<0.001**	2.09	[0.59, 6.62]	0.266
not so good	2.15	[1.53, 99.77]	**0.018**	−2.15	−4.08 [−0.56, 0.07]	**0.01**	0.23	[0.01, 0.33]	**0.001**	1.98	[0.46, 15.95]	0.268
poor	12.34			−2.32			0.07			2.72		
**Obesity**												
not applicable	1.0 → (ref)			1.0 → (ref)			1.0 → (ref)			1.0 → (ref)		
present	1.02	[0.59, 1.77]	0.938	−0.9	[−2.17, 0.38]	0.168	0.62	[0.36, 1.07]	0.086	0.63	[0.22, 1.78]	0.379
**Coronary artery disease/prior heart attack**												
not applicable	1.0 → (ref)			1.0 → (ref)			1.0 → (ref)			1.0 → (ref)		
present	5.58	[0.68, 45.54]	0.108	0.81	[−3.19, 4.82]	0.689	0.61	[0.15, 2.46]	0.489	6.51	[1.30, 32.65]	**0.023**
**High blood pressure**												
not applicable	1.0 → (ref)			1.0 → (ref)			1.0 → (ref)			1.0 → (ref)		
present	1.4	[0.88, 2.24]	0.154	0.55	[−0.49, 1.59]	0.296	0.48	[0.30, 0.75]	**0.001**	2.37	[1.34, 4.21]	**0.003**
**Diabetes**												
not applicable	1.0 → (ref)			1.0 → (ref)			1.0 → (ref)			1.0 → (ref)		
present	0.97	[0.40, 2.35]	0.938	1.39	[−1.36, 4.14]	0.32	0.92	[0.37, 2.27]	0.857	0.39	[0.05, 2.99]	0.368
**Osteoarthritis**												
not applicable	1.0 → (ref)			1.0 → (ref)			1.0 → (ref)			1.0 → (ref)		
present	1.44	[0.66, 3.14]	0.365	0.4	[−2.26, 3.06]	0.766	1.11	[0.51, 2.43]	0.796	4.88	[2.11, 11.25]	**<0.001**
**Spondylarthritis**												
not applicable	1.0 → (ref)			1.0 → (ref)			1.0 → (ref)			1.0 → (ref)		
present	1.45	[0.83, 2.54]	0.195	0.15	[−1.92, 2.23]	0.883	0.44	[0.26, 0.76]	**0.003**	1.22	[0.53, 2.80]	0.641
**Back pains**												
not applicable	1.0 → (ref)			1.0 → (ref)			1.0 → (ref)			1.0 → (ref)		
present	1.67	[1.13, 2.47]	**0.01**	−0.68	[−1.83, 0.47]	0.245	0.8	[0.55, 1.16]	0.239	2.56	[1.57, 4.17]	**<0.001**
**Rheumatic disorder**												
not applicable	1.0 → (ref)			1.0 → (ref)			1.0 → (ref)			1.0 → (ref)		
present	3.2	[0.90, 11.42]	0.073	0.55	[−0.34, 1.44]	0.226	1.7	[0.54, 5.38]	0.366	1.6	[0.34, 7.64]	0.553
**Allergy**												
not applicable	1.0 → (ref)			1.0 → (ref)			1.0 → (ref)			1.0 → (ref)		
present	1.07	[0.78, 1.48]	0.672	1.06	[0.19, 1.92]	**0.017**	1.15	[0.82, 1.60]	0.424	1.93	[1.21, 3.10]	**0.006**
**Cancer**												
not applicable	1.0 → (ref)			1.0 → (ref)			1.0 → (ref)			1.0 → (ref)		
present	1.19	[0.33, 4.23]	0.791	1.7	[−0.64, 4.04]	0.154	0.41	[0.11, 1.45]	0.165	0.91	[0.11, 7.46]	0.93
**Respiratory system diseases**												
not applicable	1.0 → (ref)			1.0 → (ref)			1.0 → (ref)			1.0 → (ref)		
present	1.91	[0.93, 3.90]	0.077	−0.07	[−1.34, 1.20]	0.914	0.51	[0.26, 0.99]	**0.045**	1.54	[0.57, 4.17]	0.395
**Depression**												
not applicable	1.0 → (ref)			1.0 → (ref)			1.0 → (ref)			1.0→ (ref)		
present	1.87	[1.04, 3.34]	**0.036**	0.69	[−0.47, 1.84]	0.242	0.88	[0.51, 1.51]	0.634	2.39	[1.17, 4.89]	**0.017**
**SARS-CoV-2 test result (COVID-19 infection)**												
negative	1.0 → (ref)			1.0 → (ref)			1.0 → (ref)			1.0 → (ref)		
positive	1.88	[1.17, 3.01]	**0.009**	0.31	[−0.27, 0.88]	0.289	0.83	[0.53, 1.30]	0.419	0.79	[0.43, 1.47]	0.462

The bold is used for significant values (*p* < 0.05).

## Data Availability

The datasets used and/or analyzed during the current study are available from the corresponding author on reasonable request.

## Supplementary Materials

The following are available online at www.mdpi.com/article/10.3390/ijerph19031299/s1, File S1 The study questionnaire.
