# Supplementary material for: Assessment of the Impact of the COVID-19 Pandemic on the Pro-Health Behavior of Poles
Source: Int J Environ Res Public Health. 2022 Jan 24;19(3):1299. doi: 10.3390/ijerph19031299 (PMC8835032; doi:10.3390/ijerph19031299)
Supplement: Supplementary file 1 [file ijerph-19-01299-s001.zip › ijerph-1540352-supplementary.pdf]

Supplementary Material File S1.

Impact assessment of the COVID-19 pandemic on the health related behavior of Poles.

We encourage everyone over the age of 18 to complete this questionnaire

Impact assessment of the COVID-19 pandemic on the health related behaviour of citizens of the European Union countries

Dear Sirs,

Internal Medicine, Metabolic Disorder and Arterial Hypertension Department and Clinic as well as the Pharmaeconomics and Social Pharmacy Centre of the Poznan University of Medical Sciences are surveying the public opinion pertaining to the impact of the COVID-19 pandemic on the health related behaviour of Poles.

The survey aims to collect information necessary for a scientific analysis and to prepare a strategy for dealing with health threatening situations.

The survey is safe, free of charge and anonymous. Personal data of respondents shall not be recorded. Data which could be used at a later date to identify the survey participants shall not be used.

In agreeing to take part in the survey you will be asked to respond to questions in an on-line survey which we will use to collect the required demographic information, assess COVID-19 contact history, COVID-19 knowledge and preventative means as well as availability of health related information with regard to COVID-19. We will also assess the impact of the pandemic on your health related behaviours.

Every respondent may approach the authors for information regarding the survey questions and to obtain a report once the survey has ran its course.

Consent to take part in the survey

I have read and understood the information pertaining to the aforementioned survey. By responding to this questionnaire I voluntarily consent to participating in the survey and I am aware that at any time I can withdraw my consent to participate in further parts of the survey without providing a reason.

Pursuant to the Polish law (Personal Data Protection Act of 10 May 2018), this survey shall be anonymous .

1. Sex: *
- female
- male

2. Age: *
- 18-24
- 25-40
- 41-60
- more than 60.

3. Education: *
- primary
- middle school
- basic vocational
- high school
- university

4. Your place of residence: *
- village
- town, up to 50 thousand residents
- town, up to 100 thousand residents
- city, up to 250 thousand residents
- city, more than 250 thousand residents.

5. What is your professional activity status? *
- Pupil/student
- Professionally active
- Unemployed
- Pensioner
- On benefits.

6. If you are professionally active, what type of work do you do? *
- Blue-collar
- White-collar

7. If you are professionally active, where do you work during the pandemic? *
- At a workplace
- I work from home
- Currently I am not working as my workplace has closed down / I have closed down my business
- Currently I am not working as my workplace has closed down / I have closed down my business, and I am afraid of losing my job
- I have just lost my job / closed my business because of the market situation brought on by the pandemic.

8. If you are professionally active, then is there an increased COVID-19 infection risk where you work? *
  - Yes, the risk is very high
- Yes, the risk is high
- Yes, the risk is moderate
- Yes, the risk is low
- There is no such risk

9. How would you describe your current state of health? *
- Excellent
- Very good
- Good
- Not so good
- Poor

10. Which chronic illness have you been diagnosed with (select as many as required) *
  - Obesity
  - High blood pressure
  - Angina Pectoris (coronary heart disease)
  - Prior heart attack
  - Prior stroke
  - Diabetes
  - Chronic obstructive pulmonary disease (COPD)
  - Osteoarthritis
  - Spondyloarthritis
  - Rheumatoid arthritis

- Airborne or food allergy
- Cancer
- Asthma
- Depression
- I have not been diagnosed with any diseases

11. Are you taking any dietary supplements? *

- Yes
- No

12. What dietary supplements are you currently taking? You may select more than one answer.

- vitamin D3 – dosage: 1000j -2000j-4000 j
- vitamin C
- group B vitamins
- zinc
- magnesium
- iron

13. Other dietary supplements: what?

…….

14. What are you guided by when selecting dietary supplements? You may select more than one answer. *

- your own experience
- pharmacist's recommendation
- ingredients
- brand name
- affordable price
- television adverts

15. Comparing with the amount which you were spending on dietary supplements before the pandemic, currently you are spending: *

- less
- the same
- more

16. Please select the statements which describe your eating habits. A multiple choice question. *

- I eat a lot of fruits and vegetables
- I eat 5 meals every day
- I eat regularly
- I drink at least 1.5 litres of water a day
- I am trying to cut down on sweets
- I avoid sparkling soft drinks
- I do my best to eat a balanced diet
- I eat a lot of fast foods
- none of the above

17. Do you use stimulants? If yes, what are you using? You may select more than one answer. *

- no
- yes, alcohol
- yes, tobacco
- yes, drugs

18. Has your diet changed because of the COVID-19 pandemic? You may select one or more answers. *

- yes, I am trying to eat a more healthy det
- yes, I have given up using stimulants
- yes, it is less healthy now
- it has not changed

19. Do you exercise? If so, how often? *

- no
- yes, once per week
- yes, 2-3 times a week
- yes, more than 3 times a week

20. Has the frequency with which you exercise changed because of the COVID-19 pandemic? *

- yes, now I exercise more
- yes, now I exercise less
- no

21. Did you have a flu jab this year? Please select one answer that best describes your circumstances. *

- yes, I also had a flu jab last year
- yes, but I did not have a flu jab in 2019
- no, but I did have a flu jab in 2019
- no, because I could not purchase a vaccine anywhere
- no, because I am not interested in getting jabs
